# *Fasciolopsis buski* (Digenea: Fasciolidae) from China and India may represent distinct taxa based on mitochondrial and nuclear ribosomal DNA sequences

**DOI:** 10.1186/s13071-017-2039-2

**Published:** 2017-02-22

**Authors:** Jun Ma, Miao-Miao Sun, Jun-Jun He, Guo-Hua Liu, Lin Ai, Mu-Xin Chen, Xing-Quan Zhu

**Affiliations:** 1 State Key Laboratory of Veterinary Etiological Biology, Key Laboratory of Veterinary Parasitology of Gansu Province, Lanzhou Veterinary Research Institute, Chinese Academy of Agricultural Sciences, Lanzhou, Gansu Province 730046 People’s Republic of China; 2 College of Animal Science and Technology, Anhui Agricultural University, Hefei, Anhui Province 230036 People’s Republic of China; 3 College of Veterinary Medicine, Hunan Agricultural University, Changsha, Hunan Province 410128 People’s Republic of China; 4 National Institute of Parasitic Diseases, Chinese Center for Disease Control and Prevention, Shanghai, 200025 People’s Republic of China; 5 Jiangsu Co-innovation Center for the Prevention and Control of Important Animal Infectious Diseases and Zoonoses, Yangzhou University College of Veterinary Medicine, Yangzhou, Jiangsu Province 225009 People’s Republic of China

**Keywords:** *Fasciolopsis buski*, Mitochondrial DNA, Nuclear ribosomal DNA, Phylogenetic analysis

## Abstract

**Background:**

*Fasciolopsis buski* is a zoonotic intestinal fluke infecting humans and pigs, but it has been seriously neglected. It is yet to know whether there is any genetic diversity among *F. buski* from different geographical locations, particularly in sequences of nuclear ribosomal DNA (rDNA) and mitochondrial (mt) DNA. Therefore, we determined the sequences of partial 18S, the complete internal transcribed spacer (ITS) rDNA and the complete mt genome of *F. buski* from China, compared the rDNA and mtDNA sequences with those of isolates from India and Vietnam, and assessed the phylogenetic relationships of this fluke and related fasciolid trematodes based on the mtDNA dataset.

**Results:**

The complete mt genome sequence of *F. buski* from China is 14,833 bp, with 36 genes, including 12 protein-coding genes (PCGs), 22 tRNA genes, and two rRNA genes (*rrn*L and *rrn*S). The AT content of *F. buski* from China is 65.12%. The gene content and arrangement of the *F. buski* mt genome is similar to that of *Fascioloides magna*. Genetic distances between isolates of *F. buski* from China and India were high (28.2% in mtDNA, 13.2% in ITS-1 and 9.8% in ITS-2) and distinctly higher than the interspecific differences between *Fasciola hepatica* and *Fasciola gigantica*. The rDNA and mtDNA datasets for *F. buski* from China (isolate from pigs) and Vietnam (isolates from humans) were identical. The intergeneric differences in amino acid and nucleotide sequences among the genera *Fasciolopsis*, *Fascioloides* and *Fasciola* ranged between 24.64–25.56% and 26.35–28.46%, respectively.

**Conclusions:**

Our results indicate that *F. buski* from China and India may represent distinct taxa, while *F. buski* in Vietnam and China represent the same species. These findings might have implications for the implementation of appropriate control strategies in different regions. Further studies are needed to decode mtDNA and rDNA sequences of *F. buski* from various geographical isolates for the better understanding of the species complex of *F. buski*.

**Electronic supplementary material:**

The online version of this article (doi:10.1186/s13071-017-2039-2) contains supplementary material, which is available to authorized users.

## Background

*Fasciolopsis buski* (Lankester, 1857) Looss, 1899, the iconic species of the genus *Fasciolopsis*, is the etiological agent of fasciolopsiasis of many mammals, such as humans, pigs, monkeys, dogs and rabbits [1]. Known as the giant intestinal fluke [2], *F. buski* is one of the largest digeneans that infect humans worldwide [3] and is largely confined to Asian countries [4–6], including China [7]. The infection with *F. buski* occurs through the consumption of raw or insufficiently cooked aquatic food contaminated with the metacercariae [8, 9]. *Fasciolopsis buski* is also found in co-infection with other soil-transmitted helminths [8] especially trematodes [10]. Fasciolopsiasis may present with moderate and heavy infection in the digestive tract [11], sometimes even followed by various complications such as acute kidney injury [12], decrease of the host immune system [13], and death [6, 13–15], leading to significant losses and public health issues in endemic areas [16, 17].

Despite the severe damage and morbidity [18] caused by *F. buski*, molecular research on this species has not attracted enough attention. Incomplete sequences of the first and the second internal transcribed spacer (ITS-1 and ITS-2) regions of nuclear ribosomal DNA (rDNA) of *F. buski* were determined and deposited [15, 19]; however, the ITS-2 sequences of isolates of *F. buski* from India and Vietnam differ by 6.7% [18], a divergence that is much larger than that between *Fasciola hepatica* and *Fasciola gigantica* (1.7%) [20]. Meanwhile, as the complete mitochondrial (mt) genome sequence of *F. buski* from India has been decoded [21], we have noticed that the sequence difference in the nicotinamide dehydrogenase subunit 1 (*nad*1) gene between the *F. buski* isolates from India and Vietnam is 17.7%, which is higher than that between *Fascioloides magna* and *Fa. hepatica* (15%) [22]. Still, no information exists to illustrate the relationships between isolates of *F. buski* from India, Vietnam and mainland China, and none of the publications have explained the sequence discrepancies among these *F. buski* isolates.

The accurate identification of trematode species is relevant in relation to better understand their biology, epidemiology and ecology, and has implications for the diagnosis of infections. Genetic markers in mtDNA and rDNA have been proven effective for identifying trematode species [23, 24] that cannot be differentiated by morphological analysis, including flukes in the family Fasciolidae [10]. Here, we determined the sequences of the complete mt genome and ITS-1, ITS-2 and partial 18S rDNA sequences of *F. buski* from China, and compared these with sequences derived from isolates from India and Vietnam to test the hypothesis that the isolate of *F. buski* sampled in China may represent a genetically distinct taxon from that sampled in India.

## Methods

### Sampling and DNA extraction

Four adult fluke specimens were obtained from intestine of naturally infected pigs from Jiangxi Province, China. These flukes were identified morphologically as *F. buski* according to the existing keys [25]. All worms were washed in 0.1 M phosphate-buffered saline (PBS), pH 7.2, fixed in 70% (v/v) ethanol and preserved at -20 °C. Total genomic DNA was extracted from individual *F. buski* specimens using sodium dodecyl sulfate (SDS)/proteinase K treatment [26] and column-purification (Wizard^®^ SV Genomic DNA Purification System, Promega, Madison, USA), according to the manufacturer’s protocol.

### Amplification of 18S and ITS rDNA

The partial 18S rDNA region of each of the four *F. buski* specimens was amplified and sequenced using primers FBJ.18SF1 (forward; 5′-ACG GGG AGG TAG TGA CGA AAA A-3′) and FBJ.18SR1 (reverse; 5′-CAC CAA CCA CCG AAT CAA GAA A-3′) which were designed based on corresponding sequences available in GenBank. The ITS rDNA region, spanning part of the 18S rRNA gene, the complete ITS-1, the 5.8S rRNA gene, ITS-2, and part of the 28S rRNA gene, was amplified and sequenced using primers BD1 (forward; 5′-GTC GTA ACA AGG TTT CCG TA-3′) and BD2 (reverse; 5′-ATG CTT AAA TTC AGC GGG T-3′) [27]. Sequence comparisons of ITS among fasciolid species were also conducted.

### Long-range PCR-based sequencing of mt genome

The primers, designed based on relatively conserved regions of mtDNA sequences from *Fas. magna* and *Fa. hepatica*, were used to amplify the sequence of the entire mt genome of *F. buski* from a single specimen in 12 overlapping fragments (Additional file 1: Table S1).

PCR reactions were conducted in a total volume of 50 μl, using 25 μl PrimeStar Max DNA polymerase premix (Takara, Dalian, China), 25 pmol of each primer (synthesized in Genewiz, Suzhou, China), 0.7 μl DNA templates, and H_2_O, in a thermocycler (Biometra, Göttingen, Germany). PCR cycling conditions started with an initial denaturation at 98 °C for 2 min, followed by 18 cycles of denaturation at 92 °C for 15 s, annealing at 55–63 °C for 15 s and extension at 60 °C for 1–5 min, followed by 92 °C denaturation for 2 min, plus 22 cycles of 92 °C for 15 s (denaturation), 55–63 °C for 15 s (annealing) and 66 °C for 1–6 min, with a final extension step for 10 min at 68 °C. A negative control (no DNA) was included in each amplification run. Positive amplicons were sent to Genewiz Company (Beijing, China) for sequencing.

### Assembly, annotation and bioinformatics analysis

Sequences were assembled manually and aligned against the entire mt genome sequences of *Fas. magna* (GenBank accession No. KU060148) and *Fa. hepatica* (NC002546) using MAFFT 7.122 to infer boundaries for each gene. Amino acid sequences of 12 protein-coding genes were translated using MEGA v.6.06 and NCBI translation Table 21 (Trematode Mitochondrial Code). The tRNA genes were affirmed using the programs tRNAscan-SE [28] or by comparison with those from the *Fas. magna* and *Fa. hepatica* mt genomes. The two rRNA genes were identified by comparison with those of *Fas. magna* and *Fa. hepatica*.

A comparative analysis of the nucleotide sequences of each protein-coding gene, the amino acid sequences, two ribosomal RNA genes, 22 tRNA genes as well as non-coding regions (NCRs) among *F. buski*, *Fas. magna* and *Fa. hepatica* was conducted.

### Phylogenetic analysis

The concatenated amino acid sequences of *F. buski* mt genome from the Chinese isolate, conceptually translated from individual genes of each mt genome, were aligned with those of published mt genomes of fasciolid trematodes, including *Fa. hepatica* (NC002546), *Fasciola* sp. (KF_543343), *Fa. gigantica* (NC024025) and *Fas. magna* (KU060148). The inferred genetic relationships of these fasciolid flukes were reconstructed based on sequences of partial *nad*1 gene (488 bp), including the isolates of *F. buski* from India (SRR924085) [21], China (NC030528) and Vietnam (EF612501) [29] and those from other fasciolid flukes. The sequence of *Paramphisitomum leydeni* (KP341657) (Paramphistomatidae) served as the outgroup.

All nucleotide or inferred amino acid sequences of were aligned using MAFFT 7.122. Poorly aligned sites and divergent regions of the alignment were eliminated using Gblocks Server v. 0.91b (http://molevol.cmima.csic.es/castresana/Gblocks_server.html) using default settings, selecting the option of less strict conservation of flanking positions. The alignment was then converted into nexus format using Clustal X1.83 and subjected to phylogenetic analysis using Bayesian inference (BI). The mixed model was used in inferred amino acid matrix, while the 4by4 was used as a nucleotide substitution model in BI analysis using MrBayes 3.1.1 [30]. Four independent Markov chain were run for 10,000,000 metropolis-coupled MCMC generations, sampling trees every 1,000 generations. The first 2,500 trees were discarded as ‘burn-in’, and the remaining trees were used for calculating Bayesian posterior probabilities. The analysis was regarded as completed when the potential scale reduction factors were close to 1, and the average standard deviation of split frequencies was below 0.01. Phylograms were prepared using FigTree v. 1.42 [31].

## Results and discussion

### Comparison of 18S and ITS rDNA

The amplicons of 18S and ITS rDNA were approximately 900 bp and 2,130 bp in length, respectively. The ITS-1 and ITS-2 were 1,505 bp and 379 bp in length, respectively. The gene boundaries of ITS were inferred by comparing with those of *Fas. magna* (GenBank: EF051080). The sequences from four *F. buski* samples were identical.

The sequences of 18S amplicons of four *F. buski* specimens from China were all 99.9% similar to those from Vietnam (AY311386 and L06668) [18]. The ITS differences between isolates from Vietnam [EF612477 (ITS-1) and EF612489 (ITS-2)] and China were 0.95% in ITS-1 and 0% in ITS-2. These divergence levels support the suggestion that *F. buski* isolates from China and Vietnam represent the same species. For the ITS rDNA sequences, differences between isolates from China and India [DQ351843 (ITS-1) and DQ351841 (ITS-2)] were 3.31% (in ITS-1) and 6.75% (in ITS-2). The difference of ITS rDNA between isolates from Vietnam [EF612477 (ITS-1) and EF612489 (ITS-2)] and India were 2.36% in ITS-1 and 6.75% in ITS-2. These were higher than the interspecific differences between *Fa. hepatica* and *Fa. gigantica* (1.2% in ITS-1 and 1.7% in ITS-2) [20], but lower than intergeneric difference between *Fas. magna* and *Fa. hepatica* (7.04% in ITS-1, 13.8% in ITS-2) [22]. The results of these comparative sequence analyses suggest that *F. buski* isolates from China and India may represent distinct fluke species.

### Mitochondrial genome content, organization and annotation

**Fig. 1:**
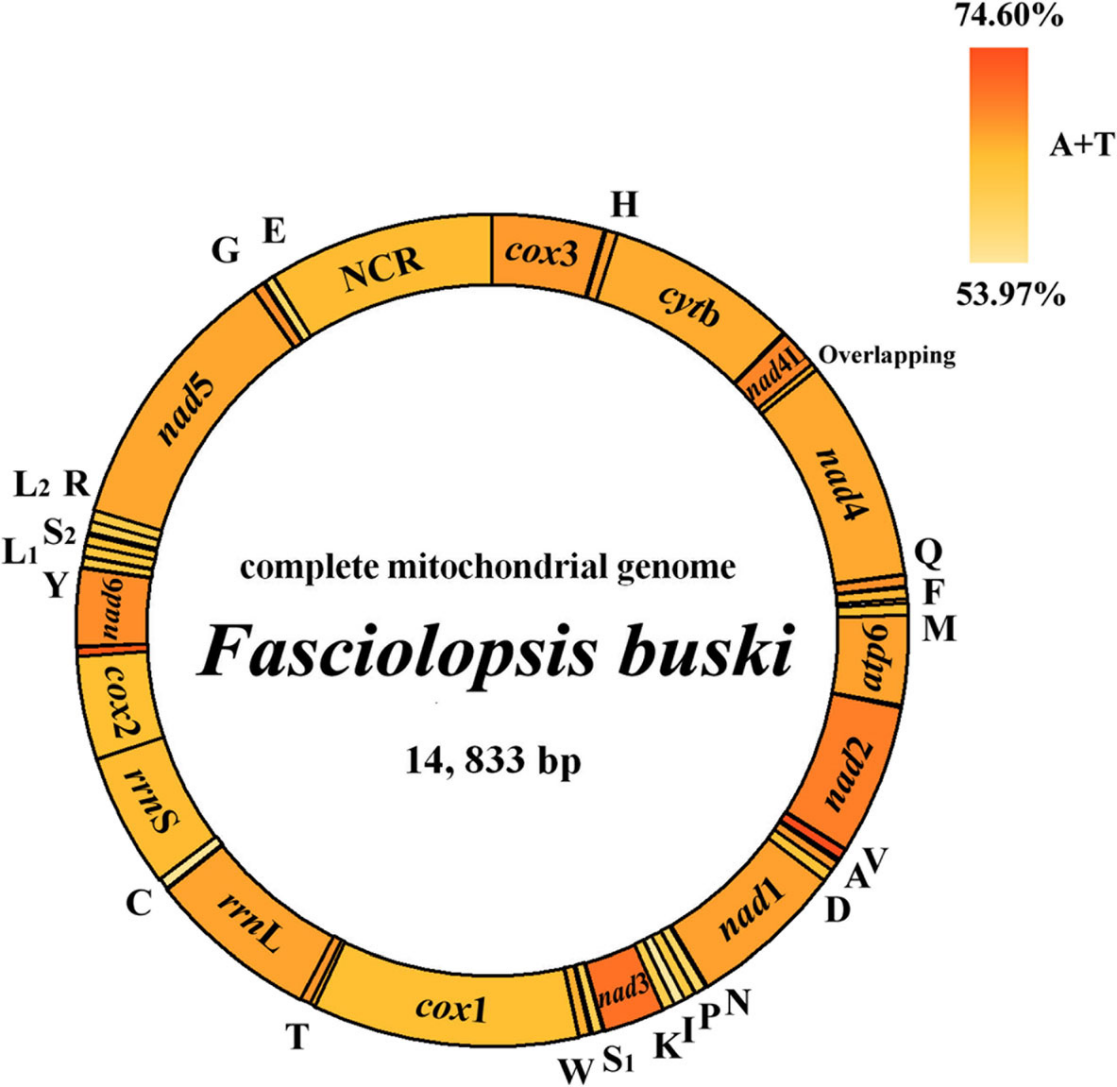
Organization of the mitochondrial genome of *Fasciolopsis buski*. The scales are approximate. All genes are transcribed in the clockwise direction, using standard nomenclature. “NCR” refers to the only non-coding region in *F. buski* data. The A + T content is shown for each gene or region of the mt genome and represented by colour

The complete mt genome sequence of *F. buski* from China (GenBank accession No. NC030528) is 14,833 bp in length (Fig. 1), and contains 36 genes, including 12 protein-coding genes (*atp*6, *cyt*b, *cox*1–3, *nad*1–6 and *nad*4L), 22 tRNA genes and two rRNA genes (*rrn*L and *rrn*S) (Table 1). The gene content and organization of *F. buski* mt genome is consistent with other fasciolid flukes [22]. There is only one NCR in *F. buski* mt genome, which is located between *trn*E (13,458–13,519) and *cox*3 (1–645) (Table 1), which is consistent with *Fas. magna* mt genome [22], whereas two non-coding regions exist in the mt genomes of *Fasciola* spp. flukes [20, 32]. The arrangement of the genes in *F. buski* mt genome is similar to that in *Fas. magna* [22].

**Table 1:** The features of the mitochondrial genomes of *Fasciolopsis buski*

Gene	Coding position (5′–3′)	Length (bp)	Start/Stop codons	No. of amino acids	Intergenic nucleotides
*cox*3	1–645	645	ATG/TAG	215	11
*trn*H	657–724	68			0
*cyt*b	725–1,834	1,110	ATG/TAG	370	9
*nad*4L	1,844–2,116	273	ATG/TAG	91	-40
*nad*4	2,077–3,357	1,281	GTG/TAA	427	5
*trn*Q	3,363–3,426	64			10
*trn*F	3,437–3,500	64			24
*trn*M	3,525–3,592	68			0
*atp*6	3,593–4,111	519	ATG/TAG	173	11
*nad*2	4,123–4,995	873	ATG/TAG	291	7
*trn*V	5,003–5,065	63			15
*trn*A	5,081–5,143	63			1
*trn*D	5,145–5,210	67			1
*nad*1	5,213–6,115	903	GTG/TAA	301	11
*trn*N	6,217–6,193	67			3
*trn*P	6,197–6,266	70			0
*trn*I	6,267–6,329	63			2
*trn*K	6,332–6,399	68			0
*nad*3	6,400–6,756	357	ATG/TAG	119	7
*trn*S1	6,764–6,823	60			14
*trn*W	6,838–6,902	65			3
*cox*1	6,906–8,447	1,542	GTG/TAG	514	30
*trn*T	8,478–8,537	60			-1
*rrn*L	8,537–9,525	989			8
*trn*C	9,533–9,597	64			-1
*rrn*S	9,597–10,371	775			-1
*cox*2	10,371–10,964	594	ATG/TAA	198	55
*nad*6	11,020–11,039	453	ATG/TAG	151	6
*trn*Y	11,479–11,535	57			6
*trn*L1	11,542–11,604	63			1
*trn*S2	11,606–11,665	60			16
*trn*L2	11,682–11,746	65			-3
*trn*R	11,744–11,810	67			-2
*nad*5	11,809–13,380	1,572	GTG/TAG	524	3
*trn*G	13,384–13,447	64			10
*trn*E	13,458–13,519	62			0
NCR	13,520–14,833	1,314			0

*Abbreviation: NCR* non-coding region

The value of total A + T content for *F. buski* mtDNA is 65.12%, which is slightly higher than that for *Fas. magna* (61.42%). The A + T content for each gene or region of *F. buski* mt genome ranged from 53.97% (*trn*I) to 74.60% (*trn*V). The nucleotide composition of *F. buski* mt genome is obviously biased towards A and T, with the low content of C (8.67%) and high content of T (46.71%) (Additional file 2: Table S2).

In the protein-coding genes of *F. buski* mt genome, ATG or GTG were used as start codons, and TAG or TAA were used as stop codons (Table 1). Incomplete codons were not detected in the mt genome of *F. buski* from China. The 22 tRNA genes of *F. buski* mt genome ranged from 57 to 70 bp in length, with their structures similar to those of *Fas. magna* and *Fa. hepatica* [20, 32]. The large ribosomal RNA gene (*rrn*L), 989 bp in length, is located between *trn*T and *trn*C. The small ribosomal RNA gene (*rrn*S) are located between *trn*C and *cox*2, which is 775 bp in length (Table 1).

The only NCR of *F. buski* mt genome (1,314 bp in length), is located between *trn*E and *cox*3, with the A + T content being 63.55%. It contains nine copies of a 101 nt complete direct repeats (TGA TTG TGT GTT GGA TAG GAT AGG TAT GTT GGG TAT TTG TTT TGG TGG ATT GGA TTG TGC CTA GGG GCT GAG TGT TAA TGA TAA TGG GAT GTG TAT TAT AT). Every two repeats are separated by 3 nt (GGG).

### Comparative analysis of mt genomes among *Fasciolopsis*, *Fascioloides* and *Fasciola*

The genetic distance across the complete mt genome between *F. buski* from China and India is 28.2%, which is higher than the interspecific difference between *Fa. gigantica* and *Fa. hepatica* (12.2%) [20], between *Paramphistomum cervi* and *P. leydeni* (14.7%) [23], and between *Paragonimus westermani* isolates from Korea and India (20.74%) [33].

The sequence difference of cytochrome *c* oxidase subunit 1 (*cox*1) gene between isolates from India (GenBank: SRR924085; 1,542 bp in length) and China is 11.8%, which is higher than that between *Fa. hepatica* and *Fa. gigantica* (9.13%) [20]. These comparisons suggest that *F. buski* from China may represent a distinct species.

Although no complete mt DNA data of *F. buski* from Vietnam were reported, the sequence of *nad*1 from the Chinese isolate is 98.6% similar to that from Vietnam (EF612501) [29], indicating that the isolates of *F. buski* from China and Vietnam might be the same species. It is noteworthy that the Chinese isolate of *F. buski* came from pigs [7], while the Vietnamese isolate infected both humans [13, 18] and pigs [29], suggesting a possible cross-transmission between humans and pigs.

The sequence differences in the complete mt genomes between *F. buski* and *Fas. magna* (28.46% at nucleotide level, 24.64% at the inferred amino acid level) are very close to those between *F. buski* and *Fasciola* spp. (26.35–26.51% and 25.56–25.48%, respectively). Considering the 12 protein-coding genes, sequence differences range between 16.12 and 29.36% at the nucleotide level, and between 14.44 and 32.55% at the amino acid level. For fasciolid flukes, at both nucleotides and amino acid levels, the comparisons among the mt genomes of the species showed that *cox*1 and *nad*4L are the most conserved genes while *nad*4 is the least conserved. In addition, *nad*1 and *cox*2 are conserved, whereas *nad*5 and *nad*6 are very variable (Table 2), which is in accordance with the characteristics of trematodes of the family Paramphistomatidae [23]. Nucleotide differences between *F. buski* and other fasciolid species ranged between 18.48 and 20.72% in *rrn*L, between 23.95 and 25.80% in *rrn*S, and between 22.03 and 26.54% in tRNA genes. We also found that *rrn*L is more conserved than *rrn*S, as observed in parasites of the Paramphistomatidae [23] and the Dicrocoeliidae [34].

**Table 2:** Comparison of nucleotides and predicted amino acids sequences among *Fasciolopsis buski* (FB), *Fascioloides magna* (FM) and *Fasciola hepatica* (FH)

Gene	nt difference (%)			aa difference (%)		
	FB/FM	FB/FH	FB/FG	FB/FM	FB/FH	FB/FG
*cox*3	24.46	22.33	22.17	29.91	28.97	30.37
*cytb*	20.84	19.86	20.13	18.65	22.43	21.89
*nad*4L	20.15	16.12	18.68	17.78	14.44	15.38
*nad*4	26.48	27.65	26.93	29.60	32.55	31.69
*atp*6	27.17	26.20	25.63	26.74	26.74	29.07
*nad*2	25.68	25.68	25.77	30.93	31.27	30.58
*nad*1	19.93	18.05	18.16	20.67	21.67	20.33
*nad*3	20.17	21.85	21.85	19.49	22.88	22.88
*cox*1	18.90	16.60	17.25	16.54	17.74	16.96
*cox*2	25.54	21.72	20.07	23.00	19.50	17.50
*nad*6	29.36	28.26	28.48	28.67	27.33	32.00
*nad*5	25.97	26.54	26.34	30.40	31.74	31.74
*rrn*L	20.72	20.04	18.48			
*rrn*S	23.95	25.80	25.57			
22 tRNAs	22.03	26.54	26.26			
Overall	28.46	26.35	26.51	24.64	25.56	25.48

### Phylogenetic analysis

**Fig. 2:**
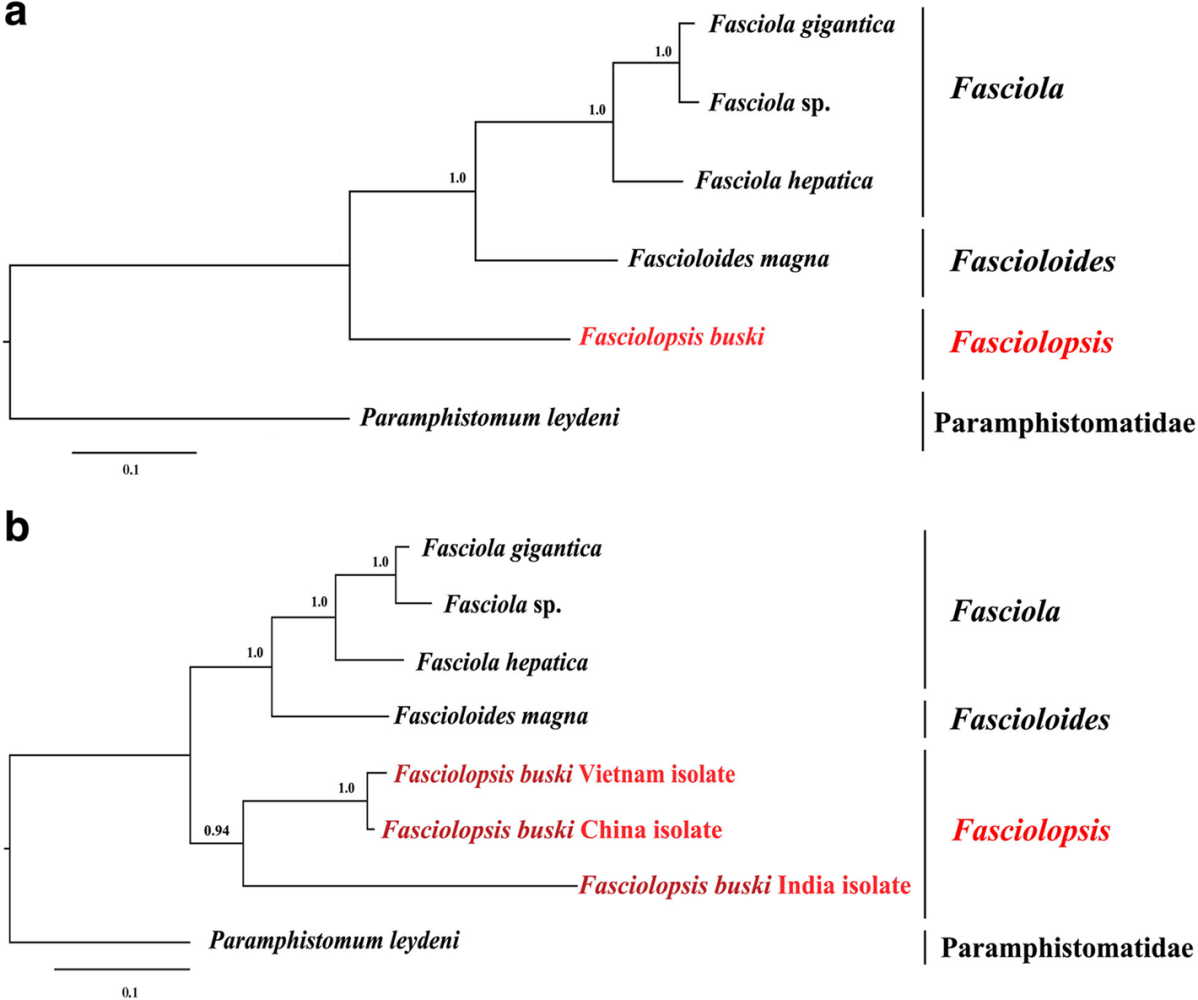
Phylogenetic relationships of *Fasciolopsis buski* and related fasciolid trematodes. Tree inferred from the concatenated amino acid sequence dataset for 12 protein-coding genes (**a**) and nucleotides for partial *nad*1 gene (**b**) from selected trematodes were constructed using Bayesian inference analysis (BI). *Paramphistomum leydeni* (KP341657) (Paramphistomatidae) served as the outgroup

The phylogenetic tree was constructed based on the concatenated amino acid sequence dataset of all 12 mt protein-coding genes of six selected trematodes (Fig. 2a). *Fasciolopsis buski* clustered with flukes of the genera *Fasciola* and *Fascioloides* within the Fasciolidae. The taxonomic status of these trematodes is in concordance with those of previous studies [20–23], with each node receiving strong nodal support (Bpp = 1).

The complete mitochondrial genome of *F. buski* from Vietnam is not decoded, only the *nad*1 gene sequence is available in GenBank. Thus, *nad*1 gene is the only mt gene with sequences available on GenBank for *F. buski* from China, India and Vietnam. Therefore, this gene was used as a molecular marker for assessing the phylogenetic relationships among species in the family of Fasciolidae. The evolutionary tree of six fasciolid species based on sequences of partial *nad*1 gene highlighted the close phylogenetic relationship between the isolates of *F. buski* from Vietnam and China, while the isolate from India appeared very distinct from these two isolates, although nodal values were marginally supported (Bpp > 0.94) (Fig. 2b).

## Conclusions

The present study determined the sequences of the complete mt genome as well as the ITS and partial 18S rDNA sequences of the giant intestinal fluke *F. buski* from China. Comparative sequence analyses indicated that *F. buski* from China and India may represent distinct taxa, while *F. buski* from Vietnam and China represent the same species. Further studies are required to better understand the *F. buski* species complex by determining the mtDNA and rDNA sequences of *F. buski* from more geographical locations.

## Additional files

Additional file 1: Table S1. Sequences of primers used to amplify fragments of *Fasciolopsis buski* mitochondrial genome. (DOCX 17 kb)
Additional file 2: Table S2. Nucleotide composition and skews of the *Fasciolopsis buski* mitochondrial protein-coding genes. (DOCX 17 kb)

## Abbreviations

BI: Bayesian inference
*cox*1: Cytochrome *c* oxidase subunit 1
ITS: Internal transcribed spacer
mt: Mitochondrial
mtDNA: Mitochondrial DNA
*nad*1: Nicotinamide dehydrogenase subunit 1
NCR: Non-coding region
PBS: Phosphate-buffered saline
rDNA: Ribosomal DNA
SDS: Sodium dodecyl sulphate
