# Supplementary material for: Fasciolopsis buski (Digenea: Fasciolidae) from China and India may represent distinct taxa based on mitochondrial and nuclear ribosomal DNA sequences
Source: Parasit Vectors. 2017 Feb 22;10:101. doi: 10.1186/s13071-017-2039-2 (PMC5322651; doi:10.1186/s13071-017-2039-2)
Supplement: Additional file 1: Table S1. — Sequences of primers used to amplify fragments of Fasciolopsis buski mitochondrial genome. (DOCX 17 kb) [file 13071_2017_2039_MOESM1_ESM.docx]

**Additional file 1: Table S1.** Sequences of primers used to amplify fragments of *Fasciolopsis buski* mitochondrial genome

| Primer | Sequence (5'-3') | Size (kb) | Amplified region |
| --- | --- | --- | --- |
| FB F1 | GTATTTGATTAGGATGGTTGAGGCT | ~ 0.6 kb | Partial *cyt*b - partial *nad*4 |
| FB R1 | GGAATCTTCGTAATAAACATCACAGC |  |  |
| FB F2 | TTTGTTGATTTGTGAGTCTCCGTATTCT | ~ 1.5 kb | Partial *nad*4 - partial *atp*6 |
| FB R2 | TAACTTACCTCTCCCCTTCCCCTCTA |  |  |
| FB F3 | TAATGTATTGTAGTTGTTTGTGTGTCTT | ~ 1 kb | Partial *nad*4L - partial *atp*6 |
| FB R3 | GAGAGAATGTTAGAAACTATAAATCAATG |  |  |
| FB F4 | TTGTTCCCTCTTATGTTTGTTTCTCG | ~ 0.5 kb | Partial *atp*6 - partial *nad*1 |
| FB R4 | TCTAACCAACTCACTCTCAGCCTCC |  |  |
| FB F5 | AGATTCGTAAGGGGCCTAAAA | ~ 0.5 kb | Partial *nad*1 - partial *nad*1 |
| FB R5 | CACGAGGTAAAGTAGCACGAGC |  |  |
| FB F6 | TATGTGAATGTAATCGTACTCCTCTTGA | ~ 2.1 kb | Partial *nad*1 - partial *cox*1 |
| FB R6 | AGAAGCAGAAAGCATAATACCAGTAACC |  |  |
| FB F7 | GGGGGTAGATTTGTTCGTATTTGG | ~ 1.7 kb | Partial *cox*1 - partial *rrn*L |
| FB R7 | TTATGTAAACCTGCTTTCATATCTTCCA |  |  |
| FB F8 | GTCAAGGTGCTGCTAATATCAAGGG | ~ 0.5 kb | Partial *rrn*L - partial *rrn*S |
| FB R8 | ACAGTCCAAAAAAACTCAACCACATC |  |  |
| FB F9 | CACAGCCCGTCACCCAGGA | ~ 0.9 kb | Partial *rrn*S - partial *cox*2 |
| FB R9 | GAAAGGCACAGAACAACACGAAAAG |  |  |
| FB F10 | GGTTATTGTACGGAGTTATGTGGTGC | ~ 0.6 kb | Partial *cox*2 - partial *nad*5 |
| FB R10 | CCCACGCACTAAACTTATACTAAACCAG |  |  |
| FB F11 | CATAATCCTAAACCGTTGCCTAAAA | ~ 1.9 kb | Partial *cox*2 - partial *nad*5 |
| FB R11 | GAAGAACATAAAAGTAACCCCTCACA |  |  |
| FB F12 | CTAGGATTTGATCTGTTGTTGTATTGA | ~ 2.5 kb | Partial *nad*5 - partial *cyt*b |
| FB R12 | GAAAATAAAAGTAACCCCTCACACAT |  |  |
