# Supplementary material for: Fasciolopsis buski (Digenea: Fasciolidae) from China and India may represent distinct taxa based on mitochondrial and nuclear ribosomal DNA sequences
Source: Parasit Vectors. 2017 Feb 22;10:101. doi: 10.1186/s13071-017-2039-2 (PMC5322651; doi:10.1186/s13071-017-2039-2)
Supplement: Additional file 2: Table S2. — Nucleotide composition and skews of the Fasciolopsis buski mitochondrial protein-coding genes. (DOCX 17 kb) [file 13071_2017_2039_MOESM2_ESM.docx]

**Additional file 2: Table S2.** Nucleotide composition and skews of the *Fasciolopsis buski* mitochondrial protein-coding genes

| Gene | Proportion of nucleotides | | | | | A + T (%) | AT Skew | GC Skew |
| --- | --- | --- | --- | --- | --- | --- | --- | --- |
|  | A | G | | T | C |  |  |  |
| *cox*3 | 17.83 | | 25.27 | 48.53 | 8.37 | 66.36 | -0.46 | 0.50 |
| *cyt*b | 16.40 | | 25.95 | 48.47 | 9.18 | 64.87 | -0.49 | 0.48 |
| *nad*4L | 19.05 | | 27.11 | 49.08 | 4.76 | 68.13 | -0.44 | 0.70 |
| *nad*4 | 15.85 | | 26.23 | 49.18 | 8.74 | 65.03 | -0.51 | 0.50 |
| *atp*6 | 16.38 | | 25.24 | 49.33 | 9.05 | 65.71 | -0.50 | 0.47 |
| *nad*2 | 14.66 | | 23.25 | 54.30 | 7.79 | 68.96 | -0.57 | 0.50 |
| *nad*1 | 15.39 | | 27.35 | 50.39 | 6.87 | 65.78 | -0.53 | 0.60 |
| *nad*3 | 16.53 | | 23.53 | 53.78 | 6.16 | 70.31 | -0.53 | 0.59 |
| *cox*1 | 16.60 | | 26.52 | 46.70 | 10.18 | 63.30 | -0.48 | 0.45 |
| *cox*2 | 23.06 | | 24.92 | 40.24 | 11.78 | 63.30 | -0.27 | 0.36 |
| *nad*6 | 16.11 | | 23.84 | 51.44 | 8.61 | 67.55 | -0.52 | 0.47 |
| *nad*5 | 16.03 | | 26.72 | 49.30 | 7.95 | 65.33 | -0.51 | 0.54 |
